# Correction: Aryl glyoxal: a prime synthetic equivalent for multicomponent reactions in the designing of oxygen heterocycles

**DOI:** 10.1039/d3ra90042h

**Published:** 2023-05-09

**Authors:** Abdur Rehman Sheikh, Anam Arif, Md. Musawwer Khan

**Affiliations:** a Department of Chemistry, Aligarh Muslim University Aligarh 202002 India musawwer@gmail.com

## Abstract

Correction for ‘Aryl glyoxal: a prime synthetic equivalent for multicomponent reactions in the designing of oxygen heterocycles’ by Abdur Rehman Sheikh *et al.*, *RSC Adv.*, 2023, **13**, 11652–11684, https://doi.org/10.1039/D2RA08315A

The authors regret that an incorrect version of product 74a in Scheme 45 was included in the original article. The correct version of Scheme 45 is presented below.

**Scheme 45:**
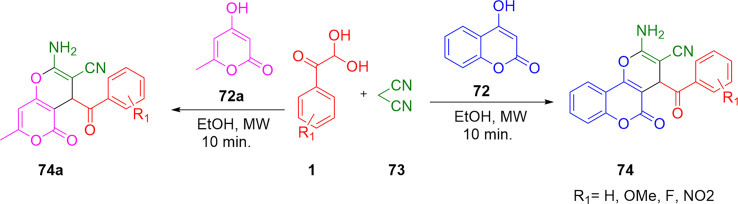
Synthesis of fused pyrans 74/74a using a microwave technique in the presence of ethanol.^1^

The authors also regret that incorrect details were given for ref. 91 in the original article. The correct version of ref. 91 is given below as ref. 2.

The Royal Society of Chemistry apologises for these errors and any consequent inconvenience to authors and readers.

## Supplementary Material

## References

[cit1] Mishra R., Choudhury L. H. (2016). RSC Adv..

[cit2] Jana A., Ali D., Bhaumick P., Choudhury L. H. (2022). J. Org. Chem..

